# iTRAQ-Based Quantitative Proteomic Analysis of the Potentiated and Dormant Antler Stem Cells

**DOI:** 10.3390/ijms17111778

**Published:** 2016-10-25

**Authors:** Zhen Dong, Hengxing Ba, Wei Zhang, Dawn Coates, Chunyi Li

**Affiliations:** 1 Institute of Special Wild Economic Animals and Plants, Chinese Academy of Agricultural Sciences, Changchun 130112, China; xi.andz@163.com (Z.D.); bahengxing@caas.cn (H.B.); zw0915@163.com (W.Z.); 2 State Key Laboratory for Molecular Biology of Special Economic Animals, Changchun 130112, China; 3 Sir John Walsh Research Institute, Faculty of Dentistry, University of Otago, PO Box 647, Dunedin 9054, New Zealand; dawn.coates@otago.ac.nz

**Keywords:** regeneration, antler stem cell, proteome, iTRAQ

## Abstract

As the only known organ that can completely regenerate in mammals, deer antler is of real significance in the field of regenerative medicine. Recent studies have shown that the regenerative capacity of the antlers comes from the pedicle periosteum and the cells resident in the periosteum possess the attributes of stem cells. Currently, the molecular mechanism of antler regeneration remains unclear. In the present study, we compared the potentiated and dormant antler stem cells using isobaric tags for the relative and absolute quantification (iTRAQ) labeling of the peptides, coupled with two-dimensional liquid chromatography-tandem mass spectrometry (LC-MS/MS) to compare the proteome profiles. Proteins were identified by searching against the NCBI nr database and our own Cervine transcriptome database, and bioinformatics analysis was conducted to identify the differentially expressed proteins. Based on this searching strategy, we identified 169 differentially expressed proteins in total, consisting of 70 up- and 99 down-regulated in the potentiated vs. dormant antler stem cells. Reliability of the iTRAQ was confirmed via quantitative real-time polymerase chain reaction (qRT-PCR) to measure the expression of selected genes. We identified transduction pathways through the Kyoto Encyclopedia of Genes and Genomes (KEGG) database, such as HIF-1 and PI3K-AKT signaling pathways that play important roles in regulating the regeneration of antlers. In summary, the initiation stage of antler regeneration, a process from dormant to potentiated states in antler stem cells, is regulated by multiple proteins and complicated signal networks.

## 1. Introduction

Regenerative medicine is a field that aims to grow back damaged/lost tissues and organs via stimulation of the body’s own reparative capability [1]. The most dramatic organ regeneration is so-called epimorphic regeneration, which represents a phenomenon of de novo development of external appendages distal to the level of amputation [2]. Deer antlers are the only mammalian appendages that can achieve complete epimorphic regeneration, and this is initiated annually from the distal ends of the permanent bony protuberances on their head know as pedicles [3]. Evidence from both histology [4] and tissue deletion experiments [5] have convincingly demonstrated that it is the distal part of pedicle periosteum (PP) that gives rise to a regenerating antler. In vitro the pedicle periosteal cells (PPCs) possess some attributes of embryonic stem cells (ESCs) as they express key ESC markers and can be induced to differentiate into multiple cell lineages; thus the PPCs are coined as the “antler stem cells” [6].

Maintenance and activation of stem cells require them to be located in a specialized microenvironment (i.e., niche), and to interact with the cells resident in the niche. Antler stem cells are no exception. Macroscopic [7] and microscopic [4] studies have revealed that the distal third of the PP and the enveloping skin are intimately bound together; whereas, the proximal two thirds of the PP and the skin are only loosely attached. Further functional analysis [8] found that the distal third of the PP regenerated an antler; whereas, the proximal two thirds PP failed to do so after separating from the enveloping skin by insertion of an impermeable membrane. All these results indicate that the mesenchymal/epithelial interactions between the PP and the enveloping skin are important for the initiation of antler regeneration. Therefore, pedicle skin may contribute to the niche important for maintenance and growth of antler stem cells. The proximal PP has been referred to as the “dormant PP” (DPP) while the distal PP has been referred to as the “potentiated PP” (PPP) [6]. Identification of the differentially expressed proteins and the activated signaling pathways in the potentiated over the dormant antler stem cells would help to unravel the underlying molecular mechanism of antler regeneration.

Over the last few years, isotope-based quantitative proteomics has become increasingly popular to overcome the disadvantages of gel-based two-dimensional electrophoresis [9]. The use of isobaric tags for relative and absolute quantitation (iTRAQ) is a well-established and validated methodology for identifying differentially regulated proteins [10]. The iTRAQ method can simultaneously analyze up to eight samples in one single test. The chemistry for iTRAQ results in the labelling of N-terminal and lysine residues, and hence tags most peptides within the samples of interest. By collision-induced dissociation or higher-energy collisional dissociation, iTRAQ reporter ions are dissociated and released in the tandem mass spectrometry (MS/MS). The intensities of the peaks are then used for the relative quantification of peptides, and hence proteins. Owing to the nature of its ultra-sensitivity and high-throughput, iTRAQ coupled with two-dimensional liquid chromatography and tandem mass spectrometry (LC-MS/MS) analysis has been deemed to be one of the most reliable methods for quantitative proteomic analysis [11,12].

The aim of the present study was to identify the differentially expressed proteins and activated transduction pathways in the potentiated antler stem cells over the dormant antler stem cells, in order to gain insights into the molecular mechanisms underlying full antler regeneration, the only mammalian example of full epimorphic regeneration. To achieve this aim, we analyzed differential expression of proteins by use of iTRAQ; we also used both the NCBI nr (non-redundant) database and our own Cervine transcriptome database to improve the identification of proteins involved in the activation of antler regeneration. Our results provided the first evidence, at the molecular level, that the potentiated antler stem cells express more proteins consistent with antler regeneration than the dormant antler stem cells, and lay the foundation for the eventual identification of the molecules that are involved in the initiation of antler regeneration.

## 2. Results

### 2.1. Identified Differentially Expressed Proteins

**Figure 1:**
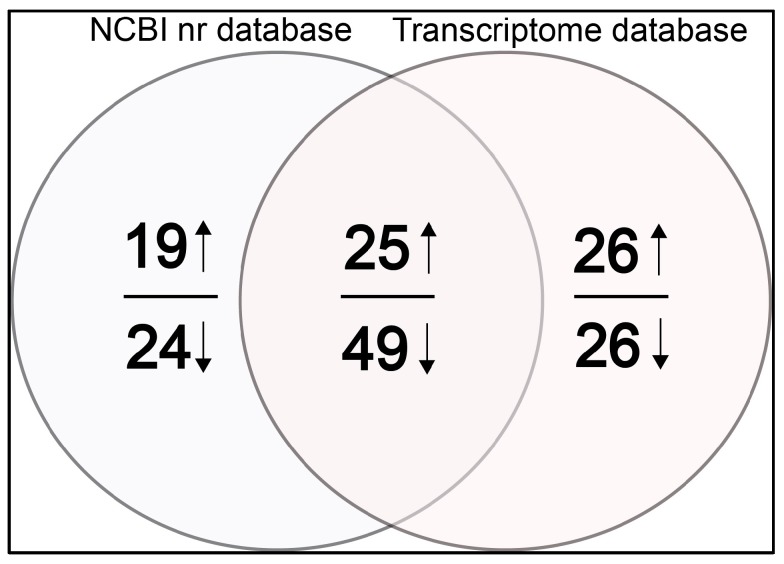
Venn diagram showing the number of total differentially expressed proteins identified by tandem mass spectrometry (MS/MS) between the NCBI nr database and transcriptome database. The overlapping regions indicate the number of shared proteins. The number above or below the horizontal line in each portion indicates the number of up- or down-regulated proteins respectively. In total, 117 and 126 proteins were identified from the NCBI nr database (Table S1) and transcriptome database (Table S2), respectively. Seventy-four proteins were identified from both databases. Among the 74 common proteins, 25 were up-regulated and 49 down-regulated. Among the 43 NCBI-specific proteins, 19 were up-regulated and 24 down-regulated. Among the 52 transcriptome-specific proteins, 26 were up-regulated and 26 down-regulated.

Using the ProteinPilot software, at the global false discovery rate of 1%, we identified 46,696 MS/MS spectra and 15,553 peptides, and detected 2500 proteins in the NCBI nr database; the corresponding numbers in the transcriptome database were 46,036, 15,361, and 2488, respectively. Finally, we obtained 169 regulated proteins in total from both the PPP cells (PPPCs) and the DPP cells (DPPCs) based on the databases of both NCBI nr (Table S1) and our own (translated from our previously published transcriptome) (Table S2). Of these 169 proteins, 43 (19 + 24) proteins were unique to the NCBI nr database; 52 (26 + 26) proteins were unique to our own database; 74 (25 + 49) proteins were found in both databases (Figure 1). Among them, 70 (19 + 25 + 26) proteins were found to be up-regulated, and 99 (24 + 49 + 26) down-regulated in the PPPCs. The advantage of our searching strategy is that it effectively increased the quantity of identified proteins compared to searching against the NCBI nr database only (Figure 1).

### 2.2. Functional Classification of the Differentially Expressed Proteins

**Figure 2:**
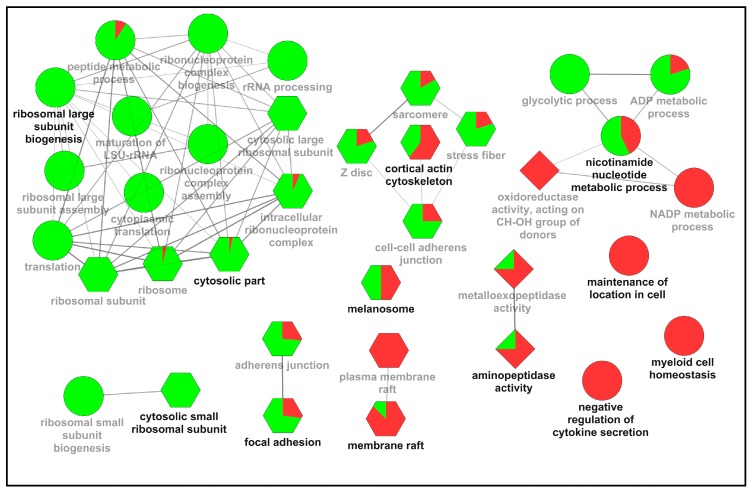
Enriched Gene Ontology (GO) network groups using ClueGO. GO categories of the identified up- and down-regulated proteins in the potentiated pedicle periosteum cells (PPPCs) are visualized as a functionally grouped network; only the terms that have *p*-value ≤0.05 are shown. Nodes in different shapes (ellipse: biological processes; hexagon: cell component; diamond: molecular function) represent specific GO terms and are grouped based on their similarity. The most significant parent or child term in each group is shown in bold, and the group is named after it. The thickness of the lines linking groups represents the value of calculated kappa score. The proportions of up- or down-regulated proteins in each GO term are indicated by red or green, respectively.

Thirty-six gene ontology (GO) terms that had *p*-values ≤0.05 were shown in the identified protein interactome (Figure 2). The most significantly enriched terms in each GO category were included for “biological process” (ellipse), “cell component” (hexagon), and “molecular function” (diamond). The identified regulated “biological process” proteins were predominantly involved in peptide metabolic process (*p*-value = 2.7 × 10^−13^) (Table S3), translation (*p*-value = 2.4 × 10^−12^), and ribosomal large subunit biogenesis (*p*-value = 4.3 × 10^−10^). Proteins involved in the “cell component” showed significant enrichment in cytosolic part (*p*-value = 5.2 × 10^−27^), ribosomal subunit (*p*-value = 1.2 × 10^−24^), and cytosolic large ribosomal subunit (*p*-value = 2.4 × 10^−24^). In addition, nearly all differentially expressed membrane-related proteins, such as the proteins locating in the (plasma) membrane raft were found to be up-regulated in the PPPCs. Proteins involved in “molecular function” were related to aminopeptidase activity (*p*-value = 3.4 × 10^−3^), oxidoreductase activity (*p*-value = 9.0 × 10^−3^), and metalloexopeptidase activity (*p*-value = 1.3 × 10^−2^); the majority of the proteins enriched in these GO terms were up-regulated in the PPPCs.

### 2.3. Enriched Pathways Participated by the Differentially Expressed Proteins

**Figure 3:**
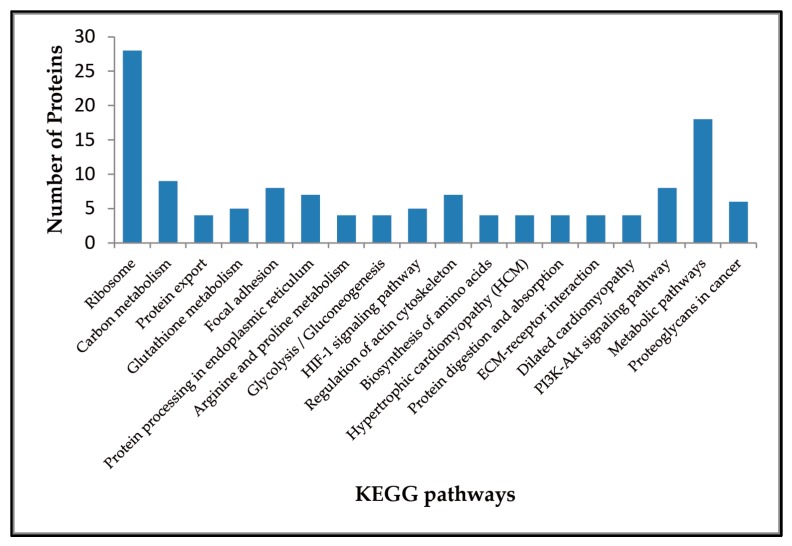
Distribution of KEGG Pathways participated by the differentially expressed proteins. KEGG pathways are arranged in ascending order according to the values of *p*-value.

One hundred and seventy one Kyoto Encyclopedia of Genes and Genomes (KEGG) pathways in total were enriched from the dataset using STRING software (Table S4). Among them, the *p*-values of 18 pathways were less than 0.01 (Figure 3). The most significant KEGG pathways for the identified proteins were Ribosome (28 proteins), Carbon metabolism (9 proteins), Protein export (4 proteins), Glutathione metabolism (5 proteins), Focal adhesion (8 proteins), and Protein processing in endoplasmic reticulum (7 proteins). Additionally, the HIF-1 signaling pathway (5 proteins), extra cellular matrix (ECM)-receptor interaction (4 proteins), and PI3K-Akt signaling pathway (8 proteins) may also be involved in potentiation process of antler stem cells.

As to the “Ribosome” KEGG pathway, all the enriched 28 proteins, such as 60S ribosomal proteins (RPL4 and RPL7, etc.) and 40S ribosomal proteins (RPS2 and RPS3A, etc.), were found to be down-regulated in the PPPCs (Table 1). Regarding “Protein export”, the majority of the enriched proteins (SPCS2, SRPRB and SEC61B) were down-regulated, but HSPA5 was up-regulated in the PPPCs. Additionally, in “Protein processing in endoplasmic reticulum”, six proteins (SAR1A, SEC61B, SEC23B, RRBP1, CRYAB, and HSP90AB1) were found to be down-regulated in the PPPCs, and only RPN2 and HSPA5 were up-regulated. In these KEGG pathways, more down-regulated proteins occurred in the PPPCs compared to the DPPCs.

**Figure 4:**
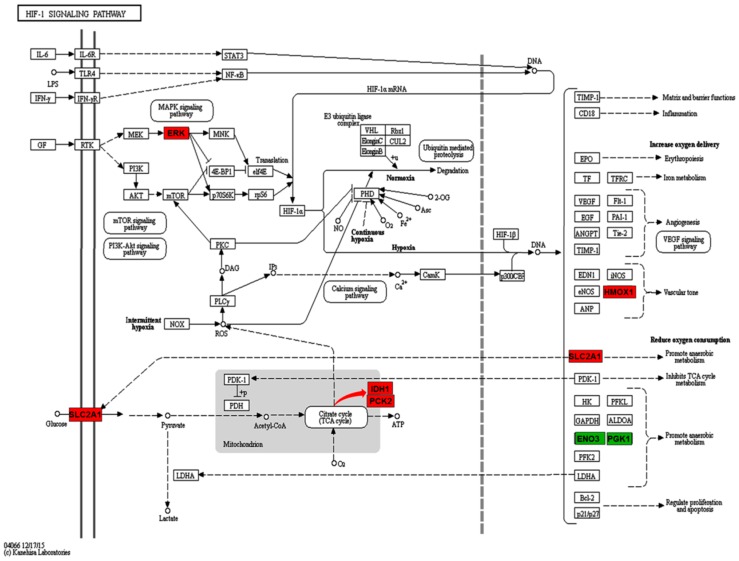
Differentially expressed proteins involved in HIF-1 signal pathway. Block in red: up-regulation; in green: down-regulation. Red arrow means that IDH1 and PCK2 are involved in the TCA cycle.

**Figure 5:**
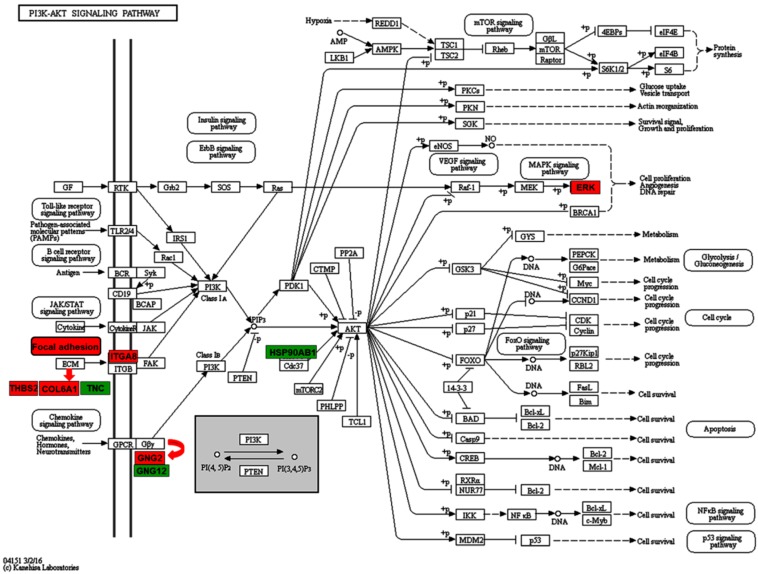
Differentially expressed proteins in PI3K-Akt pathway. Block in red: up-regulation; in green: down-regulation. Red arrow means inclusion relation (THBS2, COL6A1 and TNC are three extra cellular matrix (ECM) proteins; GNG2 and GNG12 are two G proteins).

On the contrary, in “Focal adhesion”, five proteins (THBS2, FLNB, COL6A1, ITGA8, and MAPK3) were found to be up-regulated in the PPPCs and three proteins (ACTN1, TNC, and ZYX) down-regulated (Table 1). As to the “HIF-1 signaling pathway”, there were three up-regulated proteins (SLC2A1, MAPK3, and HMOX1) and two down-regulated proteins (ENO3 and PGK1) in the PPPCs. In addition, IDH1 and PCK2, which participated in the “tricarboxylic acid (TCA) cycle” (Figure 4), were both found to be up-regulated in the PPPCs. Regarding “ECM-receptor interaction”, the majority of the enriched proteins (THBS2, ITGA8, and COL6A1) were up-regulated, and only TNC was down-regulated in the PPPCs. In “PI3K-Akt signaling pathway”, the up-regulated proteins were THBS2, GNG2, COL6A1, ITGA8, and MAPK3; the down-regulated ones were TNC, HSP90AB1, and GNG12 (Figure 5). In these KEGG pathways, more up-regulated proteins occurred in the PPPCs compared to the DPPCs.

### 2.4. Validation of the Five Selected Differentially Expressed Proteins

**Figure 6:**
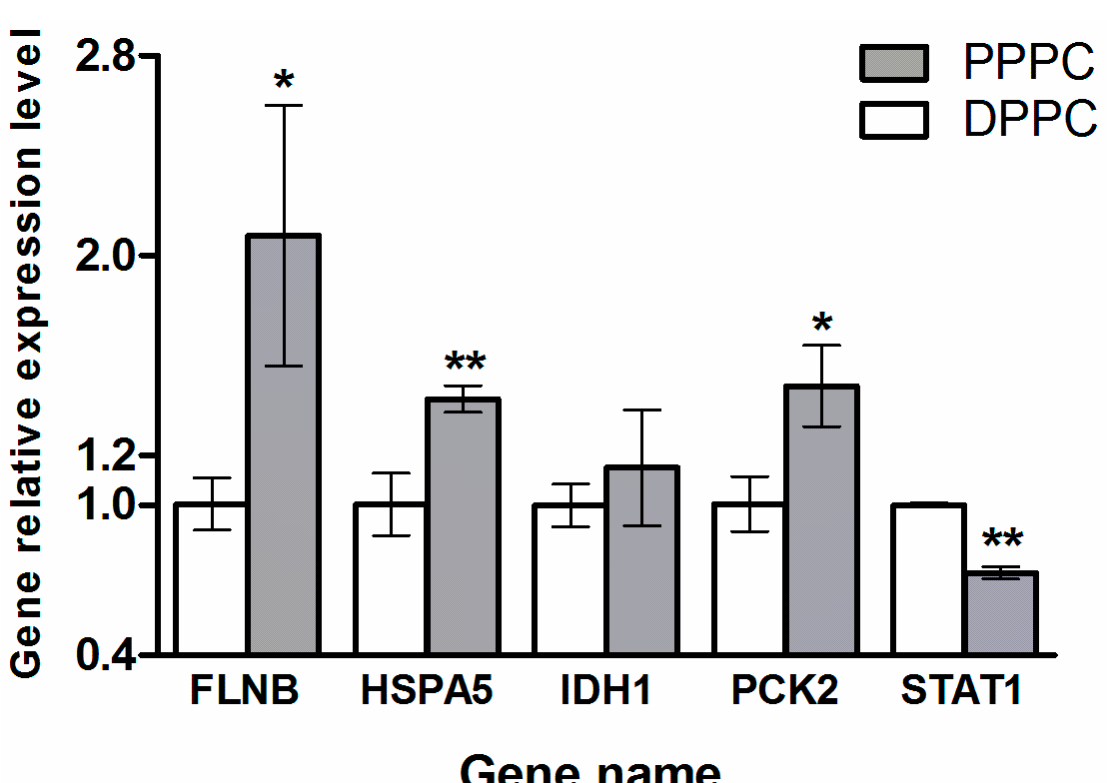
Relative expression levels of *FLNB*, *HSPA5*, *IDH1*, *PCK2*, and *STAT1* by qRT-PCR analysis, normalized to glyceraldehyde-3-phosphate dehydrogenase (*GAPDH*). The data are expressed as mean ± SD. Statistical significance: * *p*-value < 0.05 and ** *p*-value < 0.01 (the PPPCs vs. the DPPCs, *n* = 3).

Expression levels of *FLNB*, *HSPA5*, *IDH1*, *PCK2*, and *STAT1* were confirmed using qRT-PCR. Their mRNA expression levels were normalized using glyceraldehyde-3-phosphate dehydrogenase (*GAPDH*) (Figure 6). Expression of *STAT1* was found to be down-regulated, while *FLNB*, *HSPA5*, and *PCK2* were up-regulated in the PPPCs vs. DPPCs. These qRT-PCR results are consistent with those of the iTRAQ LC-MS/MS analysis. Additionally, the expression of *IDH1* was not found statistically significant in the qRT-PCR result, but the trend of its expression level in the PPPCs vs. DPPCs is consistent with that of the iTRAQ result.

## 3. Discussion

This study is the first iTRAQ-based proteomic analysis for antler stem cells (PPCs). In the study, we detected in total 169 differentially expressed proteins in the PPPCs vs. DPPCs: 70 up- and 99 down-regulated. These identified proteins were predicted to locate both in intra- and extra-cellular spaces; found to participate in multiple biological processes and signaling pathways, such as HIF-1 and PI3K-AKT. Their potential roles in antler regeneration were discussed in the following relevant parts in the discussion.

Quantitative proteomics for those species that currently lack genome sequences and intact protein databases, such as sika deer, do not normally obtain comprehensive database searching results. In order to overcome this problem and improve the search coverage, we utilized the NCBI nr database and the database created by us (defined as six-frame translation of the transcriptome of the PPPCs and DPPCs). In so doing (Figure 1), we significantly increased number of identified proteins from 117 (43 + 74) to 169 (117 + 52). Therefore, properly designing a searching strategy for genome-less species can efficiently enhance searching results.

STAT1 plays pleiotropic roles in biological processes. Loss or disruption of STAT1 results in promotion of angiogenesis [13], liver regeneration [14], myogenic differentiation [15], skeletal muscle regeneration [16], chondrocyte differentiation [17], and osteogenesis [18]. For osteogenesis, Runx2 (also known as Cbfa1) plays a central role in determining osteoblast differentiation and bone formation [19,20]. In the antler stem cells, Sun et al. [21] found that the knockdown of *Cbfa1* gene inhibited endochondral ossification of the PPCs. By binding to Cbfa1, STAT1 inhibits the process of osteoblast differentiation [18]. In the present study, STAT1 was down-regulated in the PPPCs. Therefore, less STAT1 in the PPPC would mean less Cbfa1 being neutralized. Then, it would follow that more free-Cbfa1 could be available during the process from dormant to potentiated states in the PPPCs for initiating subsequent chondrogenesis and osteogenesis during antler regeneration. The detailed mechanism needs further research.

Hypoxia is an important factor in stem cell biology. Hypoxic stress influences the physiological characteristics of embryonic stem cells [22] and neuron stem cells [23], etc. Under low oxygen tension, mesenchymal stem cells proliferate faster [24], and acquire differentiation ability toward chondrocytes [25] as well as enhanced migration ability [26]. These responses are primarily related to the HIF-1 signal pathway. HIF-1α, as a master regulator in the HIF-1 signal pathway, is directly phosphorylated and activated by one of the extracellular regulated protein kinases (ERK) MAPK3 in response to hypoxia [27]. HMOX1 is a downstream transcription factor in the HIF-1 signal pathway. Except for its vasodilatory property (Figure 4), HMOC1 also has antioxidant, anti-inflammatory, and anti-apoptotic properties [28]. In our results, ERK and HMOX1 were both found to be up-regulated in the PPPCs. Therefore, the HIF-1 signal pathway in the PPPCs may be activated by up-regulated ERK and may play a role in initiating antler regeneration by expressing downstream transcription factors, such as HMOX1. This pathway may increase proliferation and differentiation potential of the PPPCs.

Cellular respiration is a set of metabolic reactions and processes that take place in the cells to convert biochemical energy from nutrients into adenosine triphosphate (ATP); and consists of two types: aerobic (more efficient) and anaerobic (less efficient) respirations [29,30,31]. In our present study, three differentially expressed proteins (ENO3, PGK1, and SLC2A1) in the HIF-1 signal pathway were associated with anaerobic respiration (Figure 4). Among them, ENO3 and PGK1 were down-regulated, and SLC2A1 was up-regulated in the PPPCs vs. DPPCs. Besides promoting anaerobic respiration, SLC2A1 also participates in aerobic respiration (TCA cycle). In addition, two core proteins (IDH1 and PCK2) in the TCA cycle were up-regulated in the PPPCs. Overall, these results suggest that the energy metabolism in the DPPCs is anaerobic-based; whereas, in the PPPCs it is aerobic-based. If that is the case, the conversion of the cellular respiration from anaerobic to aerobic may be required during the potentiation process of antler stem cells. It would be interesting to investigate if the initiation of antler regeneration also adopts a state of aerobic respiration.

The PI3K-Akt pathway is involved in multiple cellular functions [32,33]. It plays key roles in regulating cell cycle progression, cell proliferation, differentiation, and migration [34], etc. This pathway can be activated by many types of upstream stimulators, including growth hormones, cytokines, and extra cellular matrix (ECM) proteins. In our present study, two ECM proteins (THBS2 and COL6A1) and their receptor ITGA8 were all up-regulated in the PPPCs (Figure 5). The interactions between these ligands and their receptor can activate focal adhesion kinase (FAK) protein, which may further activate PI3K class I_A_ in the PPPCs. The up-regulation of GNG2, one of the G proteins, in the PPPCs could directly activate PI3K class I_B_, and subsequently activate the PI3K-Akt pathway. At the same time, up-regulation of the ERK protein in this pathway (Figure 5) may contribute to cell proliferation, angiogenesis, and DNA repair of the PPPCs. This could be one of the reasons why the PPPCs have the potential to initiate subsequent antler regeneration. However, functions of the other two down-regulated proteins (HSP90AB1 and GNG12) in the PPPCs are thus far still unknown.

Endoplasmic reticulum (ER) plays an indispensable role in protein synthesis and secretion including the folding of protein molecules. Correct folding of newly synthesized proteins is made possible by several ER chaperone proteins, including the Hsp70 family member HSPA5. Unfolded proteins cause an unfolded protein response (UPR) as a stress response in the ER. ER stress is a state in which the folding process of proteins slows down, leading to an increase in the number of unfolded proteins [35]. In our results, HSPA5 was found to be significantly up-regulated in the PPPCs vs. DPPCs. Antlers experience an unprecedented growth rate during their growth phase, thus intense UPR process is likely to be activated in the ER of antler cells. It is understandable that HSPA5 would be highly expressed in the PPPCs, the cells that give rise to regenerating antlers, which would then help to alleviate ER stress during antler regeneration.

Filamins are involved in cell migration via formation of orthogonal networks by crosslinking with the actin cytoskeleton in cells. This network can change cell shape and improve cell motility [36]. The family of filamins consists of three proteins (FLNA, FLNA, and FLNC) [37]. Among them, FLNB is the one that expresses most ubiquitously in different tissues [38]. Knockout of FLNB is found to impair cell migration [39]. In our present study, FLNB was up-regulated in the PPPCs. Therefore, the PPPCs that are equipped with FLNA network would acquire greater motility compared to the DPPCs, which would undoubtedly facilitate migration of the PPPCs and hence the formation of an antler regeneration center.

## 4. Materials and Methods

### 4.1. Tissue Sampling and Primary Culture of the PPCs

The PP was harvested from the heads of three slaughtered three-year-old sika deer (*Cervus nippon*) in a commercial abattoir in Shuangyang nearby Changchun City under the approval from the Temporary Animal Ethics Committee of Institute of Special Wild Economic Animals and Plants, Chinese Academy of Agricultural Sciences (Permit Number: 2014–0036, date: 2014.03.15), according to the protocol described by Li and Suttie [7]. The primary culturing of the PPPCs and the DPPCs was conducted following the protocol described by Li et al. [40]. Cells were trypsinized and transferred into T75 flasks (Nest Biotechnology, Hong Kong, China) and then cultured in dulbecco modified eagle medium (DMEM) (Life Technologies, Carlsbad, CA, USA) plus 10% FBS (Gibco, Carlsbad, CA, USA), 500 U/mL penicillin, and 500 μg/mL streptomycin (Invitrogen, Carlsbad, CA, USA) for two days before being frozen. Cells were stored in liquid nitrogen in freezing medium (FBS + 10% DMSO).

The PPCs were not further purified into sub-cell populations as over 95% of these cells are known to express the embryonic stem cell marker CD9 [6]. Cells were retrieved from storage and grown in the culture medium to sub-confluence (around 85%) in T75 flasks prior to use.

### 4.2. Protein Extraction and Labeling

After discarding the culture medium in the T75 flasks, sorbitol solution (Sigma-Aldrich, Saint Louis, MO, USA) was used to rinse the cells. Subsequently, cells were trypsinized and re-suspended in the sorbitol solution, and then washed in the same solution three times. The cell pellet was re-suspended in 500 μL lysis buffer (7 mol/L Urea, 2 mol/L Thiourea, 4% 3-[(3-Cholamidopropyl)dimethyl-ammonio]-1-propane sulfonate (CHAPS), and 1% protease inhibitor cocktail) (General Electric healthcare, Fairfield, CT, USA). A Bullet Blender (Next Advance, Averill Park, NY, USA) was used to break the cells after the addition of stainless steel beads (0.5 mm in diameter; 0.5:1.0 ratios of beads). Supernatants were collected after centrifugation at 12,000 r/min and 4 °C for 5 min, and protein concentration of the supernatant was measured using the Protein Assay Kit I (Bio-Rad, Hercules, CA, USA).

An aliquot of 200 μg from each sample was mixed with 4 μg trypsin (Sigma-Aldrich, Saint Louis, MO, USA) at a final ratio of 1:50 (trypsin: sample), and incubated overnight at 37 °C. After completion of trypsin digestion, peptides were dried in a vacuum drier. Peptides were then labeled with iTRAQ reagents according to manufacturer’s instructions (Applied Biosystems, Carlsbad, CA, USA). Labeled samples were fractionated using a 4.6 mm × 250 mm Durashell-C18 column (Agela, Tianjin, China) in a RIGOL L-3000 high-performance liquid chromatography (HPLC) system (Beijing RIGOL Technology Co., Ltd., Beijing, China). After reconstitution of the labeled peptide mixture with 100 μL of buffer A (98% ddH_2_O, 2% acetonitrile, pH 10), the mixture was separated using HPLC at a flow rate of 0.7 mL/min. The elution process was done with buffer B (98% acetonitrile, 2% ddH_2_O, pH 10) as follows: 1 min 5% buffer B; 2–62 min 5%–32% buffer B; 62–64 min 32%–95% buffer B; 64–68 min 95% buffer B; 68–72 min decreasing to 5% buffer B. The elution process of fractionation was monitored by measuring the absorbance at 214 nm. Fractions were desalted with a Ziptip column (Millipore, Boston, MA, USA), and vacuum-dried.

### 4.3. Analysis of Proteins Using a LC-ESI-MS/MS

A TripleTOF 5600 coupled with an Eksigent NanoLC-2D system (Applied Biosystems) was used for protein identification and quantification. The peptide mixture was loaded into a C18 EASY-Spray column (3 μm, 12 cm × 75 μm; Agilent, Palo Alto, CA, USA) and separated using buffer A’ (100% ultrapure water, 0.1% formic acid) for 15 min at a flow rate of 2 μL/min. Peptides were eluted at 350 nL/min. The elution process was done with buffer B’ (100% acetonitrile, 0.1% formic acid) as follows: 1 min 4% buffer B’; 2–65 min 4%–32% buffer B’; 65–70 min 32%–95% buffer B’; 70–82 min 95% buffer B’; 82–85 min decreasing to 4% buffer B’; 85–90 min 4% buffer B’.

Peptides were subjected to nanoelectrospray ionization tandem mass spectrometry through a TripleTOF 5600 coupled in line to the HPLC system, with an electrospray voltage of 2.1 kV and capillary temperature of 250 °C. The analytical cycle included a MS survey scan and the scan range was 350–1800 *m*/*z*. Intact peptides were detected at a resolution of no less than 70,000 full width at half maximum (FWHM) with a 60 ms accumulation time.

### 4.4. Database Search

**Figure 7:**
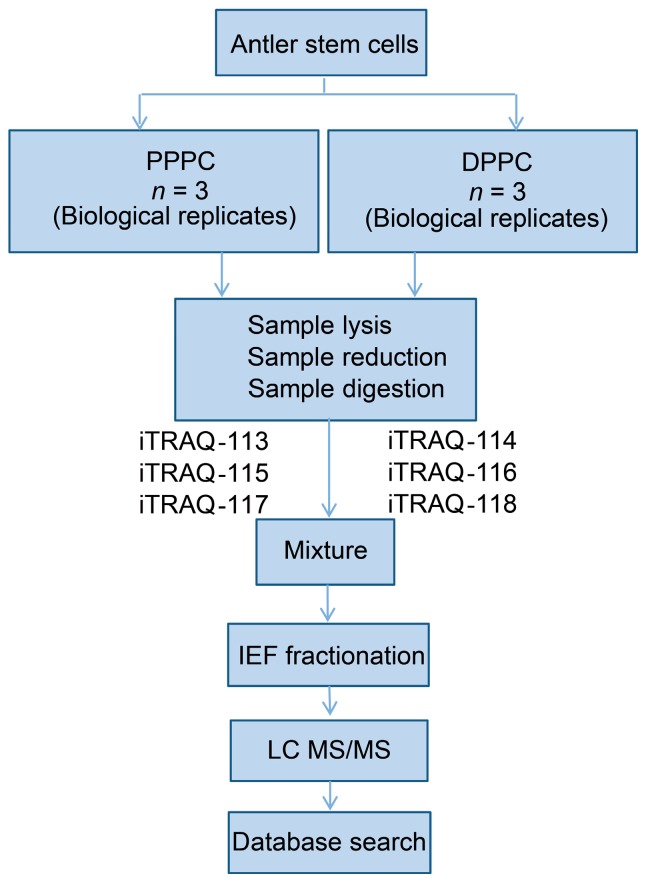
Schematic Flowchart of iTRAQ proteomics approach. Three biological replicates were used.

Quantitative proteomic analysis of sika deer peptides is limited by the availability of species specific genome or intact protein databases. In order to compensate for this, we used ProteinPilot 2.0.1 software (Applied Biosystems) to search against both the NCBI nr database (including *Homo sapiens*, *Bos taurus*, *Bos mutus*, and *Ovis aries*) as well as a database created by six-frame translation of our own PPPC and DPPC transcriptome databases (total size: 5.6 Gb; non-redundant unigene number: 63,766; N50 length: 1598 bp; average length: 886 bp). The transcriptome accession number in the Short Read Archive of the NCBI database was SRP041164. The proteome database translated from our own transcriptome and relevant annotation information can be found in the supplementary Files 1 and 2. Thresholds of confidence above 95% and global false discovery rate from fit less than 1% were firstly used for both protein identification and quantitative analysis. The following criteria were used for the identification of proteins: a mass tolerance of ±15 ppm was allowed for precursor ions and ±20 milli mass unit (mmu) for fragment ions; a maximum of two missed cleavages were allowed in the trypsin digests; cysteine carbamidomethylation (57.021 Da) was considered as a static modification; methionine oxidation (15.995 Da), iTRAQ labeling of lysines and the N-terminal amino group of peptides were set as dynamic modifications; proteins containing at least two unique peptides were used for the follow-up quantification analysis; fold change thresholds for those proteins defined as up-regulated or down-regulated proteins were a set at 1.5 (1 × 1.5 = +1.5; fold regulation calculation for those >1) or 0.67 (−1/0.67 = −1.5; fold regulation calculation for those <1); *p*-value < 0.05 was the threshold for statistical significance. The iTRAQ analysis of antler stem cells was summarized in a flow chat (Figure 7).

### 4.5. Bioinformatics Analysis

Functional classifications and enrichment analyses of Gene Ontology (GO) of the up- and down-regulated differentially expressed proteins were carried out by ClueGO (Version 2.2.5) plugin [41] in Cytoscape (version 3.4.0) [42]. Kappa statistic was utilized in the ClueGO; it was used in a similar way as described by Huang et al. [43] to connect the GO terms in the network. The ontology node shapes of “biological process”, “cell component”, and “molecular function” were ellipse, hexagon, and diamond, respectively. The following criteria were used: *p*-value for each GO term was calculated after Bonferroni step down correction and only terms with *p*-value <0.05 were shown; the network specificity was set to medium; GO term fusion was used to diminish the redundancy of the terms shared by similar associated proteins, then the most representative parent or child term was retained; the kappa score, which was calculated based on the number of proteins shared between GO terms, was set to 0.4; the organic layout (yFiles) was selected as the preferred layout, and the other parameters were set by default. STRING (version 10.0) was employed to perform a protein-protein interaction analysis based on compiled available experimental evidence and statistical enrichment tests were executed for KEGG pathway annotations [44].

### 4.6. Validation by qRT-PCR

Five differentially expressed proteins were randomly selected for further validation using qRT-PCR. Total RNA from both DPPCs and PPPCs were extracted using Trizol and purified with a silica-based spin column (Sangon, Shanghai, China) according to the manufacturer’s protocol. The reverse transcription was performed with 1300 ng of RNA/sample using a PrimeScript^TM^ First-Strand cDNA Synthesis Kit (TaKaRa, Dalian, China) following the manufacturer’s instructions. Gene-specific primers (Table S5) were designed using Primer Premier 5.0 (PREMIER Biosoft International, Palo Alto, CA, USA). Reactions were carried out using the ABI 7500 system (Applied Biosystems) with FastStart Universal SYBR Green Master (ROX) (Roche, Switzerland). *GAPDH* was used as the house-keeping gene for normalization. The qRT-PCR data were analyzed using the 2^−ΔΔ*C*q^ method. The results are presented as mean ± SD. Statistical significance was evaluated using Student’s *t*-test in GraphPad Prism 5 (version 5.01) (GraphPad Software, La Jolla, CA, USA). The *p*-value <0.05 was considered statistically significant. Results of each gene were all conducted in triplicate.

## 5. Conclusions

Overall, this is the first comprehensive study of differentially expressed proteins in the PPPCs vs. DPPCs using a quantitative proteomic approach. The first step of antler regeneration is a process from dormant to potentiated states in antler stem cells. In this study, we found this process may be regulated by multiple factors and signaling pathways. Further research is needed to validate the functions for some of the identified proteins in this study and to explore the novel molecular regulatory mechanisms underlying progression from dormant to potentiated states in antler stem cells (i.e., antler regeneration), for the only mammalian organ that can fully regenerate.

## Figures and Tables

**Table 1:** Some KEGG Pathways Participated by Differentially Expressed Proteins in the PPPCs vs. DPPCs. DPPCs, dormant pedicle periosteum cells; ECM, extra cellular matrix.

KEGG Pathway	Up-Regulated Proteins	Down-Regulated Proteins
Ribosome	–	RPL35, RPL7A, RPL29, RPL7, RPL3L, RPL19, RPL10, RPL13A, RPL8, RPL13, RPL4, RPS18, RPL34, RPS2, RPL21, RPL28, RPL35A, RPL24, RPL26L1, RPS8, RPL18A, RPL6, RPL23A, RPS13, RPL14, RPS5, RPS16, RPS27L
Protein export	HSPA5	SPCS2, SRPRB, SEC61B
Protein processing in endoplasmic reticulum	RPN2, HSPA5	SAR1A, SEC61B, SEC23B, RRBP1, CRYAB, HSP90AB1
Focal adhesion	THBS2, FLNB, COL6A1, ITGA8, MAPK3	ACTN1, TNC, ZYX
HIF-1 signaling pathway	SLC2A1, MAPK3, HMOX1	ENO3, PGK1
ECM-receptor interaction	THBS2, ITGA8, COL6A1	TNC
PI3K-Akt signaling pathway	THBS2, GNG2, COL6A1, ITGA8, MAPK3	TNC, HSP90AB1, GNG12

## Supplementary Materials

Supplementary materials can be found at www.mdpi.com/1422-0067/17/11/1778/s1.
